# Acceptability of Human Papillomavirus Vaccine: A Survey among Master of Business Administration Students in KwaZulu-Natal, South Africa

**DOI:** 10.1155/2014/257807

**Published:** 2014-08-05

**Authors:** Muhammad Ehsanul Hoque, Guido Van Hal

**Affiliations:** ^1^Graduate School of Business and Leadership, University of KwaZulu-Natal, Durban 4000, South Africa; ^2^Medical Sociology and Health Policy, University of Antwerp, Antwerp 2610, Belgium

## Abstract

Cervical cancer is a preventable public health problem. The two new human papillomavirus (HPV) vaccines are available but not accessible to everyone in South Africa, as they are very expensive. This study aimed to investigate educated peoples acceptability regarding HPV vaccination. This was a cross-sectional survey conducted among 146 master of business administration students by self-administered, anonymous questionnaire. The majority (74%) of the participants ever heard of cervical cancer, but only 26.2% heard about HPV. After reading the fact information regarding cervical cancer and HPV, the intention to vaccinate their daughters increased from 88% to 97.2% (*P* = 0.003). The majority (75.4%) indicated that HPV vaccination should be given before their daughters are mature enough to understand about sex, and 80.3% reported that they will discuss matters related to sex with their daughters if their daughters want to know about the vaccine. Those who did not want to vaccinate their daughters highlighted that they want more information regarding safety of the vaccine which might change their decision towards HPV vaccination. A health education information method can increase the vaccination acceptance rate in South Africa.

## 1. Introduction

Cervical cancer is a preventable disease, but it accounts for around 275,100 deaths, and more than half a million women are diagnosed each year in the world [1]. In South Africa, it is also the second common cancer after breast cancer. It is reported that at least 3,000 women die from cervical cancer every year, and by the year 2025, at least 4,000 women will die from cervical cancer in South Africa [2]. Because of unequal access to healthcare, differences in socioeconomic status, and exposure to HPV and HIV infection, black women are disproportionately affected [3, 4]. Cervical cancer screening in South Africa is free for women over 30 years of age as to achieve 70% coverage of this age group. But the screening in this group of women is below 20%. A recent study conducted from January 2007 to December 2010 found that annual screening coverage was between 2.9% and 4.2% [5]. Another study conducted among women who were diagnosed and managed at a Gynecological Oncology unit in South Africa reported that about 40% of women had a gynecological examination at their first visit and 15% were referred appropriately [6].

Thanks to the new development of a HPV vaccine with high efficacy, cervical cancer incidence can significantly be reduced (with 70%) if a comprehensive vaccination program could be implemented [7–9]. Many countries that have low cervical cancer rates have already implemented country wide HPV vaccination programs. But full coverage was not achieved in any country. A survey conducted in the USA in 2010 reported that 60% of parents were not interested or not sure to vaccinate their daughters [10]. School-based vaccination programs have the highest vaccination coverage in many countries like Spain, Scotland, England, and The Netherlands [11–14]. The most notable finding was that in Uganda, where a well-planned school-based campaign achieved about 95% coverage in the country [15].

The two vaccines, Cervarix (a bivalent vaccine) and Gardasil (the quadrivalent vaccine), are currently available in South Africa. But they are very expensive. The majority of the population cannot afford these vaccines for their daughters. The national Department of Health of South Africa is planning to implement HPV vaccination countrywide. Successful vaccine implementation will depend on how different population groups are aware of these vaccines.

This study investigates the acceptability of HPV vaccination among educated people like master of business administration (MBA) students in South Africa. These groups of people are holding managerial positions/owing small to medium businesses in the country. They have the capacity and capability to develop new projects towards reducing cervical cancer incidence as well as they can disseminate the information regarding HPV vaccination and cervical cancer to their colleagues or employees. Moreover, in South Africa, corporate social responsibility (CSR) is a very serious topic for discussion in the business sector. CSR means voluntary involvement, or investment, of companies in social projects that help or uplift the community in which they operate in areas such as health care, housing, education, and basic services. CSR can also be defined as the commitment by business contributing to economic development and at the same time improving the quality of life of the workforce, their families and the local community, and society at large.

## 2. Methodology

This was a cross-sectional survey study, including a fact sheet, conducted among MBA students who are currently registered at the University of KwaZulu-Natal. A total of 245 students were enrolled for the MBA program. All these students were given the possibility to participate in the study.

A self-administered questionnaire with closed and a few open-ended questions was used based on a study conducted among educated parents in India [16]. The questionnaire had three sections. The first part included sociodemographic information and the awareness regarding cervical cancer and HPV. The second section consisted of a fact sheet which provided basic information on cervical cancer, how HPV is transmitted, and efficacy and safety of the HPV vaccine. Once participants finished reading the fact sheet information they were asked to complete the third section. This section asked questions regarding parental acceptance and perception of HPV vaccination. It took about 15 minutes to complete the questionnaire.

University of KwaZulu-Natal research and ethics committee approved the study prior to data collection. Participation in the study was voluntary. Anonymity and confidentiality were maintained at all times. Prior to data collection, the main researcher communicated with the corresponding lecturers about the data collection procedure. Once the lecturers agreed, the researcher went to the corresponding lecturers' lecture room during the lecture break. The researcher then explained the aims and objectives of the study. Each participant signed a consent document before completing the questionnaire. Students who were present in the class during that day and voluntarily participated were included in the study.

Data were checked for completeness and then entered into a Microsoft Excel 2003 spread sheet and imported into SPSS 21.0. for Windows for analysis. The results were summarized using descriptive statistics: expressed as mean (SD) for continuous variables and percentages for categorical variables. Comparisons of categorical responses were evaluated using the Chi-square or Fisher's exact test. A *P* value < 0.05 was considered as statistically significant.

## 3. Results

A total of 146 students participated in the survey. Only these students were present on the day of data collection. Those who were not present had similar background characteristics because of the same admission criteria.  Table 1 summarizes sociodemographic information of the participants. Results indicated that the average age of the respondents was 34.8 years with a standard deviation of 5.9 years. More than half of the participants were males (58.9%) and Africans (55.5%) and were married (60.3%) and have a degree higher than the bachelor degree (59.3%). Regarding profession, more than two-thirds (70%) of the participants were working in a middle or senior managerial position and 77.1% were earning more than R 20,000 per month.

Participants, who had children, were asked if their children were vaccinated. Results showed that almost all the participants (90%) had given regular vaccination to their children. The most important reason to vaccinate was that vaccines prevent certain diseases (86.1%) and the main concern to vaccinate was the seriousness of the disease that the vaccine prevents. Reasons and concerns for vaccinations are shown in Table 2. Nine participants had their children not vaccinated because of not knowing about the vaccine (*n* = 5) or their physician did not advise them (*n* = 4) to have their children vaccinated.

**Figure 1:**
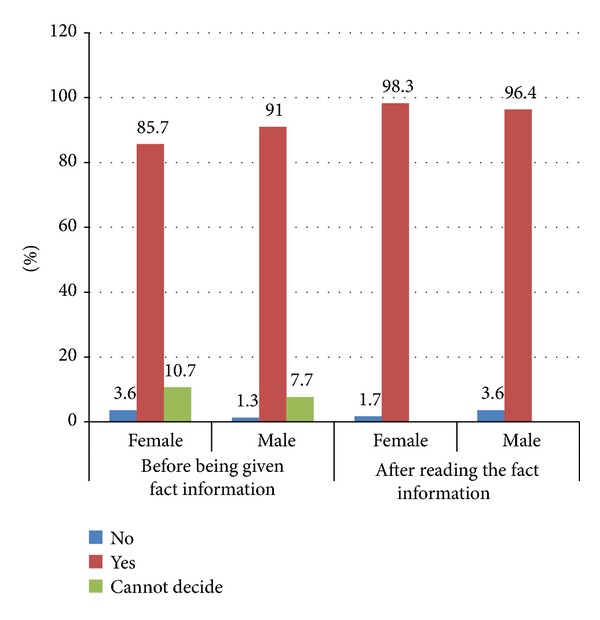
Willingness to have their daughter vaccinated against cervical cancer (%).

Overall, 74% of the participants ever heard of cervical cancer and only 26.2% heard about HPV. Initially, 88.8% of the participants were willing to have their daughters vaccinated against cervical cancer. After reading the fact sheet information regarding cervical cancer and HPV, the intention to have their daughters vaccinated significantly increased to 97.2% (*P* = 0.003) (Figure 1). Participants were also asked to indicate the best time for HPV vaccination. More than three-quarters (75.4%) indicated that HPV vaccination should be given before their daughters are mature enough to understand about sex. One out of 5 (19.6%) reported that they will wait until their daughter has grown up and can decide for herself and 5.0% suggested to have their daughters vaccinated just before they get married.

More than half (58.7%) of the participants had indicated that it is necessary to explain to their daughters before vaccination that it protects against sexually transmitted infection, whereas about two-thirds (66.0%) did not think that vaccination will send no-objection message to start sexual relationship (Table 3). The majority (80.3%) reported that they will discuss matters related to sex with their daughters if their daughters want to know about the vaccine. More than a third (35.6%) of the respondents indicated that vaccination should be given by gynecologists, 29.6% had a preference for the GP to vaccinate their daughter, 10.4% had a preference for the pediatrician, and 24.4% mentioned that it does not matter to them who gives the vaccination.

After reading the fact sheet information, four participants still did not want to give HPV vaccination to their daughters. Amongst them, three participants reasoned that the vaccine is new and they are not sure if it will be safe for their children and one participant did not give a reason. When asked what can change their mind regarding accepting the vaccine, two participants wanted a detailed report on the safety and effectiveness of the vaccine, one participant was willing to have his daughter vaccinated if it is recommended from the child's school, and the last participant did not indicate anything (data not shown).

## 4. Discussion

As far as we know, this is the first study to investigate the acceptability of HPV vaccination among educated, urban people in South Africa. Awareness regarding cervical cancer varies. This study found that a majority of the participants ever heard of cervical cancer, but only 26.2% heard about HPV. An Indian study conducted among urban, educated couples found that about a third of the participants heard about cervical cancer [16]. A cross-sectional study conducted among adults in Botswana, reported that 71% respondents heard of cervical cancer and 35% heard of HPV [17]. A Ghanaian study found that 87% of the surveyed women were aware of cervical cancer [18].

It is well known that vaccination prevents serious diseases. Globally as well as in South Africa, general vaccination coverage has increased over the last few decades. So, it was expected that all the educated people like our surveyed population vaccinated their children for their general, regular vaccination. This study found that about 90% vaccinated their daughters for general vaccination. This finding is in line with South African vaccination coverage. In South Africa, full vaccination coverage is 80% or more for one-year-old children in eight of its nine provinces [19]. Considering our educated participants, this result is low but similar to the study conducted among educated parents in India [16]. Researchers have concluded that health care workers not providing accurate information, vaccine unavailability, and lack of access to health care facilities are some of the reasons of low vaccination coverage in South Africa [20].

Globally, vaccination acceptability is high among different population groups. Studies have found high acceptability for HPV vaccination in all the countries that investigated HPV vaccination acceptability among women as well as for their daughters. Our study found that 88.8% of the participants were willing to vaccinate their daughters against cervical cancer. A recent South African study conducted among female undergraduate students found that 77.3% of the participants were willing to take HPV vaccination for themselves [21]. A study from Mali reported that 74.5% of the participants would vaccinate their children against HPV [22]. Other studies conducted from African countries reported higher acceptance rates for HPV vaccination for themselves or for their daughters despite having little knowledge regarding cervical cancer and HPV. For example, 88% of women from Botswana and 94% of Ghanaian women were willing to vaccinate themselves or their daughters [17, 18].

The effectiveness of health education on secondary prevention of cervical cancer is well documented. After reading the fact sheet information regarding cervical cancer and HPV, the respondents' intention to have their daughters vaccinated increased from 88% to 97.2%. This finding is similar to other studies conducted elsewhere. A qualitative study conducted among women aged 18–45 years in Malawi highlighted that before the study none of the participants heard of a HPV vaccine, but after given basic information, all the participants were willing to accept HPV vaccination [23]. Another study conducted in Tanzania among parents, female pupils, teachers, health workers, and religious leaders reported that after providing information about cervical cancer and HPV vaccination, most of the participants agreed that they would give HPV vaccination to their daughters [24]. A systematic review study highlighted that more information and recommendation from health care workers are the most important factors affecting parental decision making towards HPV vaccination for their daughters [25]. It is also important to note that none of the participants were undecided after reading the fact information. This suggests that health information is very important to make the desired decision.

The appropriate age to vaccinate HPV vaccines varies across different population groups. HPV vaccines are effective if they are given before the girl is not yet sexually active. In the present study, a majority mentioned that HPV vaccination should be given to their daughters before children understand about sex. A South African study reported that because of early sexual activity by the adolescents, health care providers and community respondents suggested that HPV vaccination should commence around the age of nine [26]. The present study also found similar finding as a majority of the participants mentioned that HPV vaccination should be administered before their daughters understand about sex. The study from Ghana reported that the acceptable age for HPV vaccination was 13 [18]. It was also reported that health care workers have different opinions regarding the best age for HPV vaccination. For example, one study from America found that 35% of the medical personnel would recommend HPV vaccination to 9-to-12 year-old girls, while another study reported that 77% of the paediatricians would recommend HPV vaccination to 13-to-15-year-old girls [27, 28]. Therefore, before national vaccination program implementation, health care workers need to be on the same page regarding the best age for HPV vaccination and disseminate this information to the general public.

Since HPV vaccines are new, it will face challenges to be accepted by the general population. Researchers have reported that potential side effects, health need, and benefit of the vaccine are the most imported challenges faced by the vaccine to get accepted by the general population [29]. In this study, after providing accurate information about HPV vaccines, some participants are still not happy to vaccinate their daughters. Also when asked what could be done to change their mind regarding accepting the vaccine, half of the participants required detailed information regarding safety and effectiveness of the vaccine. A qualitative study conducted in South Africa reported that some participants were concerned about vaccine safety and effectiveness, but they still supported the vaccine [26]. Among Ghanaian women, a majority were concerned about vaccine safety and side effects [18]. A Tanzanian study reported that 35% of the teachers did not want to vaccinate their daughters because of side effects and potential effects of the vaccine on future reproduction [24].

This study only sampled people from one university. As they are educated people, the results cannot be generalized. One important aspect of this study is that this group of people, who can significantly impact the society at large regarding HPV vaccination, has never been investigated before. The findings will be helpful for nationwide vaccination coverage as the business community plays a significant role in the community upliftment.

## 5. Conclusion

The present study found a high acceptability rate of HPV vaccination for their daughters in an educated group of MBA students. Educational information increased the vaccine acceptability rate. These findings are important, because due to their position in the South African society, this group of educated people can play an important role in disseminating the knowledge and attitude regarding HPV vaccination among a large part of the South African population.

## Figures and Tables

**Table 1:** Sociodemographic information of the participants.

Variables	Frequency	Percentage
Age (*n* = 141)		
30 years or below	40	28.4
31–40 years	77	54.6
>40 years	24	17.0
Average age (SD) years	34.8 (5.9) years	
Gender (*n* = 146)		
Female	60	41.1
Male	86	58.9
Race (*n* = 146)		
African	81	55.5
White	15	10.3
Colored	42	28.8
Indian	8	5.5
Marital status (*n* = 145)		
Married	88	60.3
Single	51	34.9
Living together	2	1.4
Other	5	3.4
Education (*n* = 145)		
Bachelor	59	40.7
Honors	35	24.1
Postgraduate diploma	14	9.7
Master	35	24.1
Doctorate/postdoctorate	2	1.4
Position at work (*n* = 140)		
Senior Manager	38	27.1
Middle Manager	60	42.9
Assistant Manager	18	12.9
Other	24	17.1
Monthly net income (*n* = 135)*		
<10,000	3	2.2
10,000–20,000	28	20.7
20,001–30,000	24	17.8
>30,000	80	59.3

*1 USD = 10 South African Rand.

**Table 2:** Important reasons and concerns regarding vaccination of children (*n* = 79).

	Frequency	Percent
Most important reason to vaccinate your child		
You were aware that vaccines prevent certain diseases	68	86.1
Your physician/pediatrician advised you	9	11.4
Your friends and/or relatives advised you	1	1.3
Vaccination necessary for admission to school	1	1.3
Most important concern to vaccinate your child		
The seriousness of the disease that the vaccine prevents	41	51.9
The effectiveness of the vaccine	18	22.8
Side effects of the vaccine	16	20.3
If adequate care was taken to preserve the vaccine	2	2.5
Price of the vaccine	2	2.5

**Table 3:** Participants' perception regarding HPV vaccination (%).

Statements^#^	SD	D	N	A	SA
It is necessary to explain to your daughter before vaccination that the vaccine protects against a sexually transmitted infection	22.5	7.2	11.6	26.1	32.6
Vaccination may send a no-objection message from the parents to start sexual relationships	37.7	28.3	13.0	18.1	2.9
Avoid discussing matters related to sex with your daughter if she wants to know about the vaccine/papilloma virus	45.3	35.0	10.2	5.8	3.6

^#^SD = strongly disagree, D = disagree, N = neutral, A = agree, and SA = strongly agree.
